# Virtual Training of Medical Students to Promote the Comfort and Cooperation of Patients with Neurodevelopmental Disabilities

**DOI:** 10.1007/s10803-023-05896-w

**Published:** 2023-01-17

**Authors:** Andrea Q. Hoang, Dorothea C. Lerman, Jennifer Trang Nguyen

**Affiliations:** Clinical, Health, and Applied Sciences, University of Houston-Clear Lake, 2700 Bay Area Blvd., Campus Box 245, Houston, TX 77058 USA

**Keywords:** Applied behavior analysis, Behavioral skills training, Intellectual and developmental disabilities, Medical professional training, Virtual training, Wellness examination

## Abstract

Patients with neurodevelopmental disabilities generally have less access to necessary medical care compared to those without disabilities. Barriers to adequate care include patient fear and uncooperative behavior during routine medical procedures and inadequate preparation of medical professionals to treat this population. Researchers have identified multiple behavior-analytic procedures for promoting comfort and cooperation during medical treatments. Efficient, cost-effective training programs are needed to widely disseminate behavior-analytic procedures to medical students and professionals. The purpose of this study was to assess the efficacy of a virtual training to prepare medical students to implement behavioral procedures that could be easily incorporated into typical wellness examinations. Seven medical students received behavioral skills training (BST) delivered remotely via the Internet. Results showed that the training successfully increased students’ correct implementation of the procedures in roleplay with the experimenter and with patients with neurodevelopmental disabilities. Responding also maintained at high levels 2 weeks after the training. These findings suggest that virtual BST is an efficient, practical approach for training health care professionals to implement general behavior management strategies to increase the comfort and cooperation of patients with NDD.

## Introduction

Research indicates that individuals with neurodevelopmental disabilities (NDD) generally have poorer health than those without disabilities. Individuals with NDD present with greater levels of obesity, inpatient hospitalizations, emergency room visits, and medical appointments (Boulet et al., 2009; Havercamp & Scott, 2015; Havercamp et al., 2004; Lauer et al., 2019; Weiss et al., 2018) and are less likely to receive needed medical care than those without disabilities (Center for Disease Control, 2019). Physicians also report that they order fewer preventive care services (i.e., blood pressure checks, cholesterol monitoring, mammograms) and provide less counseling on high-risk behaviors (i.e., smoking, inactivity) for patients with NDD (Rehabilitation Research & Training Center [RRTC], 2003). In fact, it has been estimated that more than a third of deaths of patients with NDD were potentially preventable by healthcare interventions (Hosking et al., 2016).

Uncooperative behavior of patients with NDD during medical appointments is one barrier to receiving adequate medical care. Patients with NDD may display uncooperative behaviors due to unfamiliarity with people in the medical environment or due to past exposure to aversive medical procedures (e.g., physical exams, blood draws) that led to a learned fear of medical stimuli (Kopecky et al., 2013). Uncooperative behaviors can take a variety of forms, including crying, protesting, refusing to follow directions, running away, and hitting others. In some cases, noncooperation may be due to poor receptive language skills (i.e., difficulty understanding instructions to open the mouth or to breathe when checking lung functioning; Cuvo et al., 2010). Uncooperative behavior may lead to the use of risky, potentially unnecessary procedures, such as restraints, sedation, or anesthesia (Boynes et al., 2010; Dougherty, 2009; Kouo et al., 2021). Such procedures may deter caregivers from seeking services for their children (e.g., Kannikeswaran et al., 2009).

Research suggests that most medical professionals do not have the knowledge and training to promote the comfort and cooperation of patients with NDD (Austriaco et al., 2019; Bruder et al., 2012; Jensen et al., 2020; Zerbo et al., 2015). As a result, medical professionals may be unwilling to implement accommodations for patients with NDD, may draw incorrect assumptions about the patients’ skills or needs, and may exclude patients with NDD from their practice due to behavioral difficulties during health care exams and procedures (Kupzyk & Allen, 2019; Nicolaidis et al., 2015; Raymaker et al., 2017; Wilson & Peterson, 2018). Medical professionals have cited lack of time, unfamiliarity with or lack of access to community resources, difficulties with communication, and lack of training or experience with this population as barriers to providing care (Carbone et al., 2010; Morris et al., 2019).

One way to reduce health inequities for patients with NDD is to provide more specialized training to medical students and practicing medical professionals (Johnson & Rodriguez, 2013). Current medical school education to work with patients with NDD typically is limited to just a few hours of didactic classroom instruction (Albino et al., 2012). Research suggests that this type of training is ineffective for preparing caregivers and professionals to work with this population (e.g., Hudson, 1982; Ward-Horner & Sturmey, 2012). A recent review of studies on autism-specific training for physicians and physician trainees also noted a lack of evidence that existing programs produce changes in physician behavior when interacting with this patient population (Clarke & Fung, 2022). Furthermore, surveys of health care providers indicate that they prefer hands-on training (e.g., Smith et al., 2021). An important consideration is how best to disseminate more specialized training to the students and professionals who would benefit. An effective training that is time- and cost-efficient would be more likely to be incorporated into existing medical school curricula or to be used by practicing professionals.

Numerous studies have demonstrated the effectiveness of behavior skills training (BST) for teaching professionals to implement behavior management strategies. BST is a competency-based training that utilizes instruction, modelling, and role-play with feedback (Parsons et al., 2012). BST has been effective to teach new skills when delivered in individual and group-training formats (e.g., Erath et al., 2020; Gormley et al., 2019; Hinkle & Lerman, 2021; Sarokoff & Sturmey, 2004). More recently, research suggests that BST also might be successful when delivered in a completely virtual format. In Matteucci et al. (2022), dental students and professionals met with an experimenter via video conferencing technology to learn how to increase the cooperation of patients with NDD during routine dental exams. After the experimenter provided initial instructions and modeling, the participants engaged in virtual role-play with the experimenter, who pretended to be a patient with NDD. The participants performed mock dental exams while practicing behavior management strategies until they mastered the strategies. Results indicated that the virtual BST was highly effective in teaching the targeted skills and took no more than 90 min for participants to complete. Most important, the participants’ skills transferred to in-person mock dental exams immediately after the training for six of the seven participants, demonstrating the generality of the fully remote training format. Virtual formats may enhance the cost-efficacy and accessibility of training, particularly if the trainer does not have to observe the trainee working with actual patients. However, further research is needed because the experimenters only assessed the skills during exams with actual patients for one of the seven participants.

Another important consideration is what to teach medical professionals as part of large-scale training programs. Ideally, the strategies should be practical and effective for increasing the comfort and cooperation of patients with NDD. The most common behavior management strategy described in pediatric and dental communities is a procedure called “tell-show-do” (Allen et al., 1990). For this strategy, the professional describes the procedure to the patient (e.g., “I am going to place this cuff around your upper arm”), shows the patient the procedure (e.g., puts the blood pressure cuff on their own arm), and then implements the procedure with the patient. Despite its popularity, tell-show-do alone is unlikely to promote cooperation in patients with NDD (e.g., Mah & Tsang, 2016).

Researchers have also identified several evidence-based behavioral interventions that are effective for increasing cooperation or decreasing fear-correlated measures (e.g., crying or screaming) during health care procedures (for reviews, see Kupzyk & Allen, 2019; St. Joseph & Machalicek, 2022). These interventions include gradually exposing the patient to elements of the procedure (e.g., Cavalari et al., 2013; Grider et al., 2012; Kupzyk & Allen, 2019; Szalwinski et al., 2019), pairing preferred items or activities with putative aversive procedures (Gorski et al., 2004; Jensen et al., 2020; Nordahl et al., 2016), providing frequent breaks from the procedures (O’Callaghan et al., 2006), preventing escape from the procedure contingent on disruptive behavior (i.e., escape extinction; Szalwinski et al., 2019), and providing reinforcement for cooperation (Cavalari et al., 2013; Grider et al., 2012; Stuesser & Roscoe, 2020).

For example, Stuesser and Roscoe (2020) examined the efficacy of differential reinforcement with and without graduated exposure for increasing cooperation during simulated medical procedures (e.g., taking temperature, drawing blood). Participants were one child and three teenagers with autism spectrum disorder. Initially, the experimenter provided praise and a food reinforcer each time a participant cooperated with a procedure without engaging in disruptive behavior. If differential reinforcement alone was ineffective, the experimenters broke down the procedures associated with noncooperation into smaller sub-steps and gradually exposed the patient to an increasing number of sub-steps. It should be noted that the experimenter always provided a brief break from the procedure when the participant engaged in disruptive behavior to ensure that the treatment would be safe and practical for medical personnel to implement (i.e., the procedure did not include escape extinction). Treatment was efficacious for all participants, with three of the four requiring gradual exposure for certain procedures. Results maintained from 2 weeks to 1 year after the final treatment session.

Although research findings indicate these behavioral interventions are effective for increasing patient cooperation, experimenters rather than health care providers implemented the interventions in most studies. Furthermore, some of these interventions may not be practical or possible for health care providers to implement. For example, for graduated exposure, the experimenter implemented the intervention over multiple sessions before the participant could complete all of the medical procedures, a strategy that might be time- and cost-prohibitive for health care providers or patients (Stuesser & Roscoe, 2020; Szalwinski et al., 2019). The experimenters implemented the interventions outside the context of health care appointments (i.e., during mock exams). This is a limitation because the gains obtained in the therapy environment may not generalize to the health care setting. In addition, experimenters have implemented escape extinction (i.e., prevented escape from the medical procedure when the patient engaged in uncooperative behavior) (Cuvo et al., 2010; Iwata et al., 1990; Slifer et al., 2011). Health care providers may be unable or unwilling to implement escape extinction (Stuesser & Roscoe, 2020).

An alternative approach is to train medical professionals to apply a set of practical and potentially effective procedures that are appropriate for the medical context. For example, professionals could pair preferred items with the exam, provide reinforcement for cooperation, and break down some of the more challenging exam steps into smaller steps within the context and duration of typical medical appointments. Research suggests that an intervention combining these components would be effective for at least a portion of patients who are uncooperative with medical exams and procedures (Kupzyk & Allen, 2019). As such, it seems appropriate to include these procedures as part of large-scale training programs.

The purpose of this study was to evaluate the effectiveness of virtual training of medical professionals in how to implement evidence-based procedures that can increase cooperation and decrease disruptive behavior during routine medical exams and that can be implemented within the context of a single appointment.

## Method

### Participants

Participants were seven medical students recruited from a university-affiliated medical clinic located in a large metropolitan city in the Southwest part of the United States. The participants had little to no former training on how to manage the behavior of patients with NDD during medical examinations and little to no experience with applied behavior analysis. All but one participant (Lulu) completed a 4-week clinical rotation at a medical clinic for adults with NDD during this study. Research sessions were integrated into the activities of the clinical rotation. Lulu was a medical student who volunteered to participate in the study at the clinic. Additional information about the participants can be found in Table 1.

**Table 1:** Participant background demographics

@Participant	Age	Race/ethnic identity	Year in medical school	# years working with patients with NDD	# years working with patients without NDD	Exposure to tell-show-do (Ranked 1–7)	Knowledge of ABA (Ranked 1–7)
Poppy	26	White	4th	< 1	4	4	3
Leona	24	Asian-Indian	4th	0	2	4	3
Darius	26	Hispanic	4th	4	3	1	1
Fiora	24	Asian	4th	1	3	4	4
Irelia	28	White	4th	1 wk	4	7	4
Zeri	26	White	3rd	< 1	3	1	1
Lulu	23	White	2nd	0	1	2	2

Likert scale for exposure to tell-show-do (1) no exposure, (4) moderate exposure (briefly discussed), (7) high exposure (specific training). Likert scale for knowledge of ABA (1) no knowledge, (4) moderate knowledge (briefly discussed), (7) high knowledge (implemented ABA practices

Nine patients from the university-affiliated medical clinic were recruited for the pre- and post-training assessments. These patients were attending the clinic for wellness check-ups and other medical issues (e.g., rashes). They ranged in age from 19 to 50 years and were diagnosed with a variety of disabilities, including autism, intellectual disabilities, and cerebral palsy. All patients were described as experiencing “anxiety” during wellness exams and had reportedly engaged in some physical resistance (e.g., pulling away) during prior exams. All sessions with patient involvement were based on the availability of the experimenter and appointments at the university-affiliated medical clinic.

### Settings and Materials

Baseline, post-training, and maintenance sessions occurred in an examination room at the medical clinic. The examination room contained a chair, a medical examination table, a sink, medical cabinets, necessary medical tools and supplies (i.e., gloves, stethoscope, otoscope, thermometer, blood pressure cuff), a timer, and laptops or desktop computers with a camera and microphone (this was utilized for video recording of sessions). The experimenter conducted training sessions remotely while the participants were in their own residences (e.g., apartments) or in separate, unused examination rooms at the clinic. The experimenter and participants used web-enabled laptop or desktop computers and video-conferencing software (i.e., Zoom™). Prior to the training, the experimenter asked the participants to gather the necessary medical tools or suggested materials to simulate medical tools that the participants did not possess (e.g., a pen to simulate an otoscope).

The experimenter constructed a wellness exam task analysis through consultation with medical professionals and nursing staff. The task analysis, consisting of eight separate components of a wellness exam, included 38 steps (see Table 2). The participants had access to this task analysis in all sessions of the study. The experimenter also created 20 patient histories in consultation with medical professionals from the university-affiliated medical clinic to ensure likeness to real patients. The participant received a new patient history prior to each session (see further description below).

**Table 2:** Wellness exam task analysis

Step/task		Duration
*Pre-examination steps*		
1. Medical professional greets the patient		5 s
2. Patient sits in the examination chair		10 s
*Blood pressure measurement*		
1. Patient holds out arm		5 s
2. Patient tolerates blood pressure cuff to be slid around bicep		5 s
3. Patient tolerates adjustment of blood pressure cuff		5 s
4. Patient tolerates pumping of blood pressure cuff		5 s
5. Patient tolerates fully inflated blood pressure cuff and stethoscope on arm for 30 s–1 min		60 s
6. Patient tolerates removal of the blood pressure cuff		15 s
*Lung examination*		
1a. Patient tolerates placement of stethoscope on left upper chest		3 s
1b. Patient takes a deep breath		7 s
2a. Patient tolerates placement of stethoscope on right upper chest		3 s
2b. Patient takes a deep breath		7 s
5a. Patient tolerates placement of stethoscope on left upper back		3 s
5b. Patient takes a deep breath		7 s
6a. Patient tolerates placement of stethoscope on right upper back		3 s
6b. Patient takes a deep breath		7 s
7a. Patient tolerates placement of stethoscope on left lower back		3 s
7b. Patient takes a deep breath		7 s
8b. Patient tolerates placement of stethoscope on right lower back		3 s
8b. Patient takes a deep breath		7 s
*Heart examination*		
1. Patient tolerates stethoscope on right upper chest		20 s
2. Patient tolerates stethoscope on left upper chest		20 s
3. Patient tolerates stethoscope on left lower chest		30 s
*Abdominal examination*		
1. Patient lays down		10 s
2. Patient tolerates stethoscope on stomach and visual inspection		10 s
2. Patient tolerates right-side upper palpations		10 s
3. Patient tolerates left-side upper palpations		10 s
4. Patient tolerates left-side lower palpations		10 s
5. Patient tolerates right-side lower palpations		10 s
*Mouth/throat examination*		
1. Patient opens mouth		5 s
2. Patient tolerates tongue pushed down by depressor		5 s
3. Patient tolerates otoscope in mouth during observation		15 s
4. Patient tolerates palpation on throat		15 s
*Eye examination*		
1. Patient looks straight		2 s
2. Patient tolerates otoscope nearby right eye with light on during observation		5 s
3. Patient tolerates otoscope nearby left eye with light on during observation		5 s
*Ear examination*		
1. Patient tolerates otoscope in right ear during observation		30 s
2. Patient tolerates otoscope in left ear during observation		30 s

### Response Measurement and Interobserver Agreement

Trained observers collected data live via videoconferencing sessions and/or from video-recorded sessions. Observers used paper and pencil to score correct or incorrect implementation of tell-show-do, noncontingent reinforcement, graduated exposure, and consequences for cooperation and disruptive behavior during each opportunity within a session (see Table 3 for descriptions of the procedures). A response was only considered correct if it occurred within 10 s of the opportunity; a response was scored as not applicable if there was no opportunity to perform the response. Some responses could only be implemented once during each session (e.g., the first and second responses in Table 3), whereas others had multiple opportunities within and across exam steps. For each exam, the experimenter calculated the number of responses implemented correctly divided by the total number of opportunities and multiplied by 100. During the simulated patient sessions, the research assistant followed prepared scripts to ensure that the number of opportunities for each response was consistent across sessions.

**Table 3:** General behavior management strategy steps

Step #	Task
1	Participant greets the patient
2	Participant provides noncontingent access to a caregiver-reported highly preferred item and does not remove for the entire examination
3	Participant labels the tool and shows it to the patient
4	Participant explains why they are using the tool with a gesture to the terminal body part
5	Participant implements the medical examination procedure
6	Following completion of a procedure, the participant provides specific praise
7	Following completion of a procedure, the participant provides a 15 s break from the examination
8	Following disruptive behavior, the participant provides a 5 s break from the examination
9	The participant repeats the procedure after the 5 s break
10	The participant terminates the procedure following the second occurrence of disruptive behavior and implements the next step of the medical examination
11	Participant implements graduated exposure as first step for high priority procedures or those with a history of problem behavior
12	Participant places the tool 6 in away from the terminal body part for 5 s
13	Participant places the tool 3 in away from the terminal body part for 5 s
14	Participant moves the tool to the terminal body part for 1 s
15	Participant adjusts the tool (if applicable)
16	Participant completes ¼ of the procedure
17	Participant completes ½ of the procedure
18	Participant completes the procedure

During sessions with actual patients, observers collected data on the occurrence of patient cooperation and problem behavior to determine the correct implementation of the behavior intervention components by the participants. None of the patient participants engaged in problem behavior during the medical exams. Cooperation with a step in the medical exam was scored if the participant permitted the medical professional to complete the step as indicated on the task analysis.

A second observer collected data during at least 25% of the sessions for each participant for the purpose of calculating interobserver agreement (IOA). The experimenter compared data collected by the primary and secondary observers on an opportunity-by-opportunity basis. The experimenter scored agreement as both observers scoring the same response (correct or incorrect), divided the sum of agreements by the total number of opportunities, and converted to a percentage. Mean IOA was 92.0% for Poppy (range, 85.4–99.7%), 97.3% for Leona (range, 94.6–100%), 89.6% for Darius (range, 84.9–98.5%), 91.3% for Fiora (range, 84.1–98.4%), 89.3% for Irelia (range, 78.9–100%), 94.4% for Zeri (range, 88.1–100%), and 98.2% for Lulu (range, 93.1–100%).

### Procedures

All participants experienced baseline sessions with simulated patients (research assistants who role played as a patient), virtual training sessions, and post-training sessions with simulated patients. Due to limits on participant availability and COVID-10 pandemic restrictions, only a portion of participants also experienced baseline sessions with actual patients, post-training sessions with actual patients, and maintenance sessions with simulated or actual patients. As noted previously, the experimenter implemented all sessions within the context of a 4-week clinical rotation at the medical clinic (exception was Lulu; see further information below). Due to variations in participant availability and other factors, the scheduling of sessions across the 4 weeks differed across participants. The schedule for each participant is shown in Table 4.

**Table 4:** Participant session schedules

Participant	Week 1	Week 2	Week 3	Week 4	Week 5
Poppy	T(BL) TH (TR) F(PT)			T (MN)	
Leona	T(BL) TH (TR) F(PT)			T (MN)	
Darius	T (BL) F (TR; PT)			T (MN)	
Fiora	T (BL) F (TR)			T (PT)	
Irelia	T (BL) F (TR; PT)			F (MN; PM)	
Zeri	T (BL; PBL) TH (BL) F (TR; PT)	TH (PPT)		TH (MN)	
Lulu	T (BL; PBL) F (TR; PT; PPT)	T (PPT)			T (MN)

*T* Tuesday, *TH* Thursday, *F* Friday; In parenthesis: *BL* baseline, *TR* training, *PT* post training, *MN* maintenance, *PM* patient maintenance, *PBL* patient baseline, *PPT* patient post training

#### Baseline (Simulated Patient)

The experimenter scheduled a 90-min in-person appointment with each participant to conduct baseline sessions in the absence of the other participants. At the start of each baseline session, the experimenter stated, “Complete a wellness visit check-up with the information provided,” and presented written instructions stating that they had 15 min to conduct a medical wellness exam. The instructions included a patient history, the reason for the visit (e.g., earache) or a high-priority portion of the exam (e.g., checking the heart), the topography of problem behavior previously seen during medical examinations (if any), and the task analysis of the medical exam procedures. The session began when the participant vocally indicated that they were ready to begin the exam. The participant had access to the written instructions throughout each session. The experimenter followed prepared scripts to role play as a simulated patient. The scripts determined when the experimenter engaged in problem behavior, cooperation, and resistance while allowing the procedure to be completed. If the participant asked a question, the experimenter referred them to the written instructions and said that any remaining questions would be addressed later. The experimenter did not provide additional feedback or guidance. Sessions were terminated after 15 min or if the participant stated that they were done with the exam.

#### Baseline (Actual Patient; Zeri and Lulu Only)

The participant conducted a wellness exam under the supervision of a physician from the clinic with a patient recruited from the clinic. Only two participants had an opportunity to experience this condition due to pandemic-related restrictions in effect at times during the course of the study. The experimenter provided the same instructions and materials as those in the baseline sessions with the simulated patient. However, the supervising physician discussed the patient’s medical history, the reason for the visit or the high priority portion of the exam, and the topography of problem behavior previously seen during medical examinations (if any) with the participant. As noted previously, patients who were involved in this study were described as having anxiety and some physical resistance to wellness exams (e.g., pulling away). No additional instruction or feedback on behavioral management techniques was provided during or after the session. The supervising physician provided feedback to the participants about medical-related issues and procedures.

#### Virtual Training

The participants received virtual training individually (Irelia and Zeri) or in groups of two (Poppy and Leona, Darius and Fiora, Lulu and a research assistant acting as a participant). The location of the participants and number of participants in each session was based on their schedule and availability. Participants were either located in their residence or in separate rooms at the clinic during the training. This was determined based on the university schedule and whether the students were required to be at the clinic on the morning of the scheduled training. The participants could take notes and ask questions throughout the training. The experimenter first provided a 90-min narrated Microsoft PowerPoint® presentation with information on the research and science of applied behavior analysis, a rationale for the behavioral procedures and a description of the recommended procedures to increase cooperation and decrease problem behavior. Following the presentation, the participants watched an 18-min video that showed the experimenter modeling the procedures while completing an exam with a simulated patient.

Next, each participant role played virtually with the experimenter, who acted as a patient, while the other participant observed (if another participant was present). Prior to the first role play, the experimenter explained how to simulate implementation of the procedures with the simulated patient. The experimenter demonstrated how to gesture towards the camera or bring tools close to the camera while interacting with the simulated patient. The participant then practiced all of the behavioral procedures with the exception of graduated exposure while completing two medical exam procedures on the exam task analysis (e.g., listening to the heart; looking into the ears). During each role play, the experimenter followed a different simulated patient script and provided specific praise and corrective feedback after each exam procedure. The participant continued to receive feedback after each exam procedure until they had completed a full medical examination with 100% accuracy. At that point, the participant continued to practice while the experimenter provided feedback after the completion of three exam procedures until the participant responded correctly on 100% of the opportunities.

Next, the participant implemented graduated exposure for one medical exam procedure during each role play exam using the steps and performance criterion described previously. Once they implemented the behavioral procedures correctly on 100% of opportunities, the participant practiced implementing a full wellness exam with all behavioral procedures while receiving experimenter feedback. Practice continued the participant implemented the behavioral procedures correctly on at least 85% of opportunities. At this point, the other participant in the training (if a second one had been observing) practiced implementing the behavioral procedures in role play with the experimenter exactly as described previously. The other participant observed this training.

Following training, the experimenter provided the participants with a copy of the handout outlining the behavior management strategies described and the 18-min training video and recommended that each participant refer to these materials before they treat future patients who have a history of noncooperation or problem behavior during medical exams.

#### Post-Training (Simulated Patient)

The experimenter conducted these sessions between 1 and 24 h following the virtual training session for all participants except Fiora, who could not participate due a required period of quarantine following exposure to COVID-19 (see Table 4). Procedures were identical to those in the simulated-patient baseline. The purpose was to evaluate the effects of the training on the participant’s accuracy in implementing the behavioral procedures in person rather than through a virtual modality. Sessions continued until the participant implemented the procedures correctly on at least 90% of opportunities for two consecutive sessions.

#### Post-Training (Actual Patient; Zeri, Lulu, and Irelia)

Only three participants had the opportunity to conduct post-training sessions with actual patients due to pandemic-related restrictions in effect during the study. The experimenter arranged for these sessions to occur within 4 days of the initial training based on the availability of the patients at the medical clinic. One participant (Irelia) conducted a session with a patient 2 weeks following the training due to scheduling difficulties. Procedures were identical to those in the actual-patient baseline condition with one exception. Immediately following the patient exam, the experimenter met with the participant to provide an opportunity for the participant to describe any modifications that they had deliberately made to the instructed procedures and the rationale for doing so. If the participant correctly vocalized any steps omitted and provided an appropriate rationale for doing so, the step was omitted when scoring correct responses.

The following were accepted omissions to the behavior management strategy steps: (a) the participant did not implement noncontingent reinforcement (step 2) because the caregiver could not identify any preferred items for the patient; (b) the participant did not include a gesture in step 4 because the patient had visual impairments or the participant believed that vocal instructions would be sufficient given the patient’s receptive language skills; (c) the participant did not implement steps 8–10 because the patient did not emit any problem behavior during the appointment; and (d) the participant did not implement graduated exposure (steps 11–17) because the caregiver could not identify a specific part of the exam that had previously evoked problem behavior or resistance from the patient.

The experimenter provided no other feedback on behavior management strategies. If the participant did not perform the procedures correctly on at least 90% of opportunities with two consecutive patients, the experimenter provided specific feedback consisting of praise for correct responses and corrective feedback for incorrect responses. Zeri was the only participant who met the criterion for feedback. Following sessions with feedback, the experimenter arranged for Zeri to conduct another exam with a patient. Procedures were identical to those of the post-training with patients.

#### Maintenance (Simulated Patient)

The experimenter evaluated maintenance of the skills 2 weeks following training. Procedures were identical to those in the post-training (simulated patient) condition.

### Social Validity Measure

The experimenter provided each of the participants with a modified version of the Treatment Acceptance Rating Form (TARF) questionnaire (Reimers & Wacker, 1988). The participants completed the questionnaire immediately after the post-training session and then again at the end of the maintenance session. The second questionnaire was identical to the first one but included two open-ended questions. The participant was asked “If you did implement the behavioral techniques with patients on your rotation, approximately how many patients?” and “If you did **not** implement the behavioral techniques with any patients on your rotation, what are the reasons that you did not?” Participants completed these questionnaires and submitted them in person or via email to the experimenter.

### Experimental Design

The experimenters used a nonconcurrent, multiple baseline design across participants to evaluate the effectiveness of the virtual training on participants’ acquisition, generalization, and maintenance of the behavioral procedures.

## Results

**Fig. 1:**
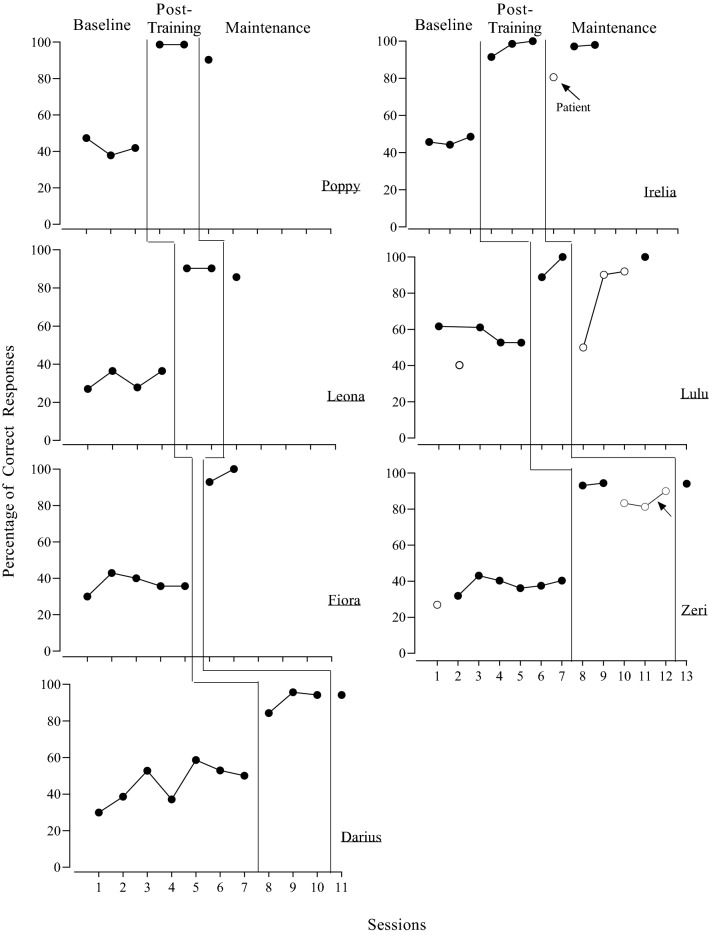
Results for each participant. The arrow indicates when Zeri received additional feedback

Results for all participants are shown in Fig. 1. Poppy’s correct responding during baseline (simulated patient) averaged 42.3% of opportunities. During post-training (simulated patient), Poppy’s correct responding increased to a mean of 98.6% of opportunities with a slight decrease to 90.3% during maintenance (simulated patient). Leona’s correct responding during baseline (simulated patient) averaged 32% of opportunities. Leona’s correct responding was high during post-training (simulated patient), averaging 90.3% of opportunities. Like Poppy, correct responding slightly decreased during maintenance (simulated patient) to a mean of 85.7% of opportunities. Fiora’s correct responding during baseline (simulated patient) averaged 36.7%. Fiora was not able to participate in the post-training phase due to COVID-19 restrictions. However, during maintenance (simulated patient), Fiora responded correctly on a mean of 97% of opportunities. Darius’s correct responding during baseline (simulated patient) averaged 45.7%. During post-training (simulated patient), Darius’s correct responding averaged 91.4%. His performance remained high during maintenance (simulated patient; M = 94.2%).

Irelia’s correct responding averaged 46.7% of opportunities during baseline (simulated patient) and increased to a mean of 96.7% of opportunities during the post-training phase (simulated patient). During maintenance, Irelia responded correctly on 80.6% of opportunities with an actual patient. Her most frequent error was failing to provide praise following the patient’s cooperation with a medical procedure. Due to scheduling issues, Irelia could not participate in any further sessions with actual patients. However, she responded correctly to a mean of 97.6% of opportunities during subsequent sessions with simulated patients.

Lulu’s correct responding during baseline (simulated patient) averaged 57.1%. She responded correctly on 40.3% of opportunities during a baseline exam with an actual patient. Lulu’s correct responding during post-training (simulated patient) increased to a mean of 94.5% of opportunities. During her first post-training (patient) exam, Lulu responded correctly on 50% of opportunities. During this first patient exam, Lulu saw a patient who had been diagnosed with cerebral palsy and was minimally responsive. During the post-session debrief with the experimenter, Lulu stated that during the appointment she did not provide praise or vocally state the instruction or tool being used due to the patient’s lack of responses. However, during the meeting, Lulu corrected herself and stated that she should not have assumed the patient’s level of understanding and she should still implement those two steps of the procedure; these responses indicated self-correction without any feedback from the experimenter. She then implemented the procedures with high fidelity following this self-correction. During the next two post-training (patient) exams, Lulu’s correct responding exceeded the mastery criterion of 90%. During maintenance (simulated patient), Lulu correctly responded on 100% of opportunities.

Zeri’s correct responding during baseline (simulated patient) averaged 36.6% of the opportunities. She responded correctly on 26.9% of opportunities during a baseline exam with an actual patient. Zeri’s correct responding during post-training (simulated patient) increased to a mean of 93.87% of opportunities. Her performance decreased somewhat during post-training exams with actual patients. She responded correctly on a mean of 82.3% of opportunities because she often failed to provide praise or a 15-s break following the patients’ cooperation with the medical procedure. Because Zeri’s performance did not meet the mastery criterion of 90% or above for two consecutive sessions, the experimenter provided feedback to Zeri about her performance with the second patient. Following feedback, Zeri responded correctly on 90% of the opportunities during an exam with a third patient. During maintenance (simulated patient), Zeri responded correctly on 94.1% of opportunities.

All participants met the mastery criterion during a remote training that lasted no more than 3 h, with each participant requiring no more than four practice trials with each set of procedures (e.g., noncontingent reinforcement) and, on average, about 30 min of practice to meet the criterion. Finally, because the participants’ opportunities to implement different components of the behavioral procedures with actual patients (as opposed to simulated patients) depended on the patients’ behavior, the data were further analyzed to identify the procedures that participants implemented during these exams. This information is displayed in Table 5.

**Table 5:** Components scored during patient generalization probes

Participant	Phase	Components implemented from general behavior management strategy steps (Table 3)										
		1	2	3	4	5	6	7	8	9	10	11–18
Irelia	Maintenance	x		x		x	x	x				
Zeri	Baseline	x	x	x	x	x	x	x				
	Posttraining	x		x		x	x					
	Posttraining	x	x	x	x	x	x	x				
	Posttraining	x	x	x	x	x	x	x				
Lulu	Baseline	x	x	x	x	x	x	x				
	Posttraining	x	x	x	x	x	x	x				
	Posttraining	x		x	x	x	x	x				

Overall, results from the social validity survey completed post training and at maintenance indicated that the medical students found the behavioral procedures to be acceptable, that they believed that the procedures would be effective with patients, and that they would be willing to implement the procedures with patients (see Table 6 for social validity ratings). On the survey completed at maintenance, participants estimated that they had used the procedures with a range of 2 to 30 patients (M = 10.5) during their rotation at the clinic. Furthermore, the participants provided similar ratings on the survey immediately following the training and after implementing the procedures with patients (see Table 7 for social validity ratings of individual participants). Anecdotally, many of the participants stated that they wished this training was a part of their standard curriculum, and one participant expressed an interest in providing the training to her other classmates.

**Table 6:** Mean participant ratings on the social validity survey administered at post-training and maintenance

Items	Avg	Range
1. How clear is your understanding of the behavioral techniques? (1 = not at all clear; 7 = very clear)	6.6	6–7
2. How acceptable do you find the techniques to be regarding your concerns about your patients? (1 = not at all acceptable; 7 = very acceptable)	6.5	6–7
3. How willing are you to carry out these techniques? (1 = not at all willing; 7 = very willing)	6.6	5–7
4. How reasonable do you find these techniques to be? (1 = not at all reasonable; 7 = very reasonable)	6.4	5–7
5. How costly will it be to carry out these techniques? (1 = not at all costly; 7 = very costly)	1.7	1–3
6. How likely do you think there might be disadvantages in following these techniques? (1 = unlikely; 7 = very likely)	2.7	1–5
7. How likely are these techniques to make improvements in your patient’s behavior? (1 = unlikely; 7 = very likely)	5.8	4–7
8. How much time will be needed for you to carry out these techniques? (1 = little time will be needed; 7 = much time will be needed)	5	4–7
9. How confident are you that the techniques will be effective? (1 = not at all confident; 7 = very confident)	6	5–7
10. How willing would you be to change your exam routine to carry out these techniques? (1 = not at all willing; 7 = very willing)	5.9	3–7
11. How often did you implement the techniques during your rotation? (1 = not at all; 7 = often)	6.2	6
12. If you did implement the behavioral techniques with patients on your rotation, approximately how many patients?	10.5	2–30
13. If you did **not** implement the behavioral techniques with any patients on your rotation, what are the reasons that you did not?	—	—

Ratings for each question were averaged across the post-training and maintenance social validity surveys

**Table 7:** Participant ratings for each question on the social validity survey administered at post-training and maintenance

Participant	Phase	Question #									
		1	2	3	4	5	6	7	8	9	10
Poppy	PT	7	6	7	7	1	5	6	1	6	6
	MN	6	6	6	6	1	2	5	3	5	6
Leona	PT	6	6	6	6	1	2	6	5	6	5
	MN	6	6	7	5	1	1	5	4	5	5
Darius	PT	6	7	7	7	2	4	7	7	7	7
	MN	6	7	7	7	2	2	7	4	7	7
Fiora	PT	6	6	7	7	1	1	6	4	6	7
	MN	7	7	7	7	3	1	6	6	7	7
Irelia	PT	7	7	7	7	1	1	7	5	7	7
	MN	7	7	7	7	1	1	7	5	7	7
Zeri	PT	6	6	5	5	3	4	5	5	4	4
	MN	7	6	6	6	2	3	6	6	5	6
Lulu	PT	7	7	7	7	2	2	7	5	6	7
	MN	7	7	7	7	1	1	6	4	6	7

*PT* Post Training, *MN* Maintenance

## Discussion

Results suggest that virtual BST may be an efficient, practical approach for training health care professionals to implement a set of general behavior management strategies to increase the comfort and cooperation of patients with NDD during exams and procedures. For all participants, performance in the virtual environment generalized to an in-person format and maintained across 2 weeks. Further, results for a subset of participants suggested that the training was sufficient to prepare them to implement the strategies with actual patients in the absence of further training. This latter conclusion must remain tentative, however, because only three of the seven participants had the opportunity to apply the trained skills to actual patients during the study; furthermore, their performance with patients was somewhat variable, and none had an opportunity to implement graduated exposure or respond to problem behavior during the exams.

The study extends the current literature by evaluating the effectiveness of brief virtual BST when teaching health care students to apply a set of practical behavior management strategies that should be effective for at least a portion of patients with NDD. The majority of prior studies on autism-specific training for health care professionals evaluated lecture-based, educational programs, a format that is unlikely to produce changes in practices, and none evaluated changes in actual trainee behavior (for reviews, see Clarke & Fung, 2022; Walsh et al., 2021). Furthermore, few prior studies on BST have examined its efficacy when conducted solely in a virtual environment (see Lloveras et al., 2022, for a notable exception). The abbreviated, virtual nature of the training may increase its accessibility to health care students and professionals. All of the participants completed the training in less than 3 h, which included 90 min of didactic instruction, 18 min of video modeling, and practice in role play (approximately 30 min per participant). Furthermore, the two formats embedded within the training (hands on and web based) were consistent with the reported preferences of medical professionals (Bruder et al., 2012; Smith et al., 2021).

Typically, medical students receive no more than brief didactic classroom instruction on treating patients with NDD (Austriaco et al., 2019). Nonetheless, voluminous amounts of research indicate that BST is far more likely to change actual practices. If widely available, this training might increase the number of providers who are willing and able to work with this population, directly addressing one barrier to equitable health care for individuals with NDD (Carbone et al., 2010; Morris et al., 2019). Moreover, training health care providers how to increase the comfort and cooperation of patients with NDD may decrease the number of patients who experience restraint or sedation during health care exams and procedures, as well as those who require more intensive services from behavior specialists.

The social validity questionnaire focused on the content of the training rather than on the training modality. However, results indicated that the participants generally found the behavior management strategies to be acceptable and effective with patients. On the questionnaire completed 2 weeks after the training, all of the participants reported that they had applied the strategies to multiple patients during their clinical rotation. Most importantly, many of the participants rated the procedures more favorably after implementing the procedures with patients.

Nonetheless, further research is needed to address various limitations of this study. First, due to the COVID-19 pandemic and scheduling difficulties, the experimenter observed just three of the seven participants implementing the procedures with actual patients. Baseline data with an actual patient could not be collected for one of those participants (Irelia), and only one patient was available for a post-training exam with this participant. Second, the participants only had opportunities to implement a subset of the procedures with actual patients because none of the patients displayed disruptive or noncompliant behaviors during the exams; in addition, the caregivers could not report a specific part of the exam that had evoked anxiety or physical resistance in past exams. Thus, the experimenter could not observe the participants’ responses to problem behavior or their implementation of graduated exposure. Third, maintenance of the skills was limited to a 2-week period and all but one of those maintenance checks were conducted with simulated patients.

Another limitation was that the experimenter conducted training with just one or two participants simultaneously during meetings that lasted up to 3 h, a format that may not be practical for preparing large groups of medical students and professionals. Further research is needed on the efficacy of more efficient training formats. For example, instructors could provide the didactic and modeling portion of BST via a training video to large groups of trainees and then arrange short, small-group role-plays for the practice and feedback portion. Pyramidal training, or train-the-trainer arrangements, also might increase the accessibility of the training and make it more practical to embed within existing medical school curriculum or large health organizations (e.g., Mery et al., 2022). Continuing education for practicing professionals likely will need to incorporate asynchronous training modalities (i.e., on-line instructional modules and videos) to permit more flexible scheduling. These asynchronous opportunities could be combined with periodic virtual meetings to provide opportunities for discussion, role plays, and case presentations, similar to that utilized in the Extension for Community Healthcare Outcomes (ECHO) model (e.g., Mazurek et al., 2020).

A final limitation is that the procedures were not developed with the input of caregivers and patients with NDD. Obtaining more information about potential factors that might increase patient comfort during health care appointments could help improve the efficacy and acceptability of the procedures. Further research should assess the acceptability of selected behavior management strategies by soliciting input from caregivers and patients before and after they receive direct exposure to the procedures.

It also should be emphasized that this training focused on just one important component of a comprehensive curriculum for preparing health care providers to serve patients with NDD (see Clarke, 2022). It was not envisioned as a stand-alone program but as a model for disseminating knowledge and evidence-based practices when providing services to this population. Further research is needed on best practices for embedding a complete curriculum into existing training programs. In addition to the content of this training, health care providers would benefit from information that dispels misconceptions about patients with NDD, increases knowledge of their health care needs, and improves health care providers’ communication with their patients, among other topics (Havercamp et al., 2016, 2021).

In addition to changes in health care practices, further research should measure other important outcomes of specialized training programs, such as changes in the number of patients with NDD included in the trainees’ practice and the satisfaction of these patients with the services provided. Long term, it would be important to evaluate the impact of training on the health of patients with NDD and other measures of health inequities experienced by this population. With the continued rise in the estimated prevalence of NDD, particularly ASD, it is imperative that all health care providers receive the training and experience needed to serve this population effectively.
